# A two-step multiple-marker strategy for genome-wide association studies

**DOI:** 10.1186/1753-6561-1-s1-s134

**Published:** 2007-12-18

**Authors:** Hugues Aschard, Mickaël Guedj, Florence Demenais

**Affiliations:** 1 INSERM, U794, Tour Evry 2, 523 Place des Terrasses de l'Agora, 91034, Evry, France; 2 Université d'Evry Val d'Essonne, Boulevard François Mitterrand, 91025, Evry, France; 3 Serono, France; 4 Laboratoire Statistique et Génome, CNRS UMR8071, INRA U1152, Université d'Evry, Tour Evry 2, 523 Place des Terrasses de l'Agora, 91034, Evry, France

## Abstract

Genome-wide association studies raise study-design and analytical issues that are still being debated. Among them, stands the issue of reducing the number of markers to be genotyped without loss of efficiency in identifying trait loci, which can reduce the cost of studies and minimize the multiple testing problem. With this aim, we proposed a two-step strategy based on two analytical methods suited to examine sets of markers rather than single markers: the local score, which screens the genome to select candidate regions in Step 1, and FBAT-LC, a multiple-marker family-based association test used to obtain significance levels of regions at step 2. The performance of this strategy was evaluated on all replicates of Genetic Analysis Workshop 15 Problem 3 simulated data, using the answers to that problem. Overall, seven of the nine generated trait loci were detected in at least 87% of the replicates using a framework designed to handle either association with the disease or association with the severity of disease. This multiple-marker strategy was compared to the single-marker approach. By considering regions instead of single markers, this strategy minimizes the multiple testing problem and the number of false-positive results.

## Background

Genome-wide association studies with hundreds of thousands of markers (SNPs), as made possible by new high-throughput genotyping technologies, raise many study-design and analytical issues, among which the multiple testing problem occupies a central role. Several strategies have been proposed to confront this problem, including one-stage and multiple stage study designs, analytical approaches in one or multiple steps, and use of one or multiple data sets [1,2]. The two-stage study design, which consists of genotyping many markers in an initial sample at a first stage and a subset of selected SNPs in another sample at a second stage, has often been chosen for cost reasons. However, it has been shown to have other advantages because it allows the number of analyzed markers to be decreased, thus minimizing the multiple testing problem, while maintaining adequate power [3]. To further minimize the multiple testing problem, a number of methods have been proposed for the joint analysis of neighboring marker loci, including haplotype analysis and multiple regression-based methods [4]. As a result, it appears relevant to select whole genomic regions rather than single markers at a first stage of genome-wide association studies, but, to our knowledge, this has been scarcely considered until now. We are proposing a two-step strategy based on two new methods that each have the ability to examine sets of markers rather than single markers: the local score statistic, which can be used to select genomic regions based on a sequence of association signals at a first stage, and FBAT-LC (linear combination of family-based association tests) [5], which allows testing for association with sets of markers in the selected regions at a second stage. Using sums, the local score statistic identifies accumulations of high statistics in a sequence. In molecular biology, this method has been applied to the localization of hydrophobic domains in proteins and the identification of similar regions among two or more sequences [6]. It was recently applied to association studies for the detection of significant local high-scoring segments from case-control data [7]. The second method, FBAT-LC, is a new extension of FBAT for the joint analysis of multiple markers in family data that does not require haplotype reconstruction [5]. Our goal was to assess the statistical performance of the proposed two-step multiple-marker strategy by analyzing the rheumatoid arthritis (RA) case-control and affected sib-pair (ASP) simulated data (Problem 3 of Genetic Analysis Workshop 15), using the set of 9187 SNPs distributed across the genome. Our aim was also to compare this multiple-marker strategy to the single-marker based approach.

## Methods

### Two-step multiple-marker strategy

We propose a flexible multiple-marker analytical approach for genome-wide association studies made up of two steps. In the first step, the local score method is applied to case-control data in order to detect and rank candidate regions across the genome. It serves as a screening tool. In the second step, these candidate regions are tested for association with the studied phenotype in a sample of family data using FBAT-LC [5] and the *p*-values obtained are then corrected for multiple testing. Each of these two steps is independent from each other and can be modified according to the type of data collected. We chose to make full use of Problem 3 data, which included both case-control and family data, thus guiding the choice of the test statistics suited to these data.

### Step 1: Detecting candidate regions

The local score method used the Pearson chi-square statistic applied to the case-control genotypic contingency table for each marker to produce a sequence of scores [7].

Let ***X ***= (*X*_*i*_)_*i*=1,...,*n *_be a sequence of real random variables. In our context ***X ***represents a sequence of statistics of association attached to each marker along the genome. The statistic:

$$H=\underset{1\leq a\leq b\leq n}{\max}\sum_{a}^{b}X_{i}$$

defines the local score assigned to ***X***. In practice, it corresponds to the value of the region with the maximal sum of scores *X*_*i*_. Consequently, the variables *X*_*i *_must be negative on average otherwise the best region would easily span the entire sequence. This definition is restrained to the highest-scoring region. The next high-scoring ones are potentially interesting as well because the data set may contain more than one trait locus (TL). We define the *k*^th ^best region as the local score of the initial sequence disjoint from the preceding *k *- 1 best regions. In this case *H*^(1) ^> ... > *H*^(*k*) ^are the scores of the *k *first and distinct highest-scoring genomic regions. Advantages over simple-marker strategies arise from the ability of this statistic to identify a set of candidate genomic regions that may contain genes involved in the disease.

The algorithm of the local score approach includes the three following procedures: i) *producing the initial sequence ****X***: we assign to each marker a statistic of association (*X*_*i*_) corresponding, in our case, to the Pearson chi-square test of case-control marker genotype frequencies. A constraint of this strategy is to have ***X ***negative on average; that does not happen with positive statistics such as Pearson chi-square, so a constant *δ *must be subtracted from the whole signal ***X***. In this study, *δ *corresponds to the value of statistic *X*_*i *_at the classical 5% level and we let ***X' ***= ***X ***- *δ*; ii) *identifying the highest-scoring region*: a simple approach to get the local score from ***X****' *consists of comparing the value of $\sum_{a}^{b}X{'}_{i}$ for all possible regions [a; b] but excluding those regions spanning different chromosomes; iii) *identifying the next high-scoring regions *by using an iterative algorithm: find the highest-scoring region, remove it from ***X'***, and apply the algorithm again until there are no more positive local scores in the sequence. At the end, the number of tests has been reduced from *M *markers to *N *candidate genomic regions ranked according to their local scores.

### Step 2: Testing candidate regions for association

The new FBAT extension proposed by Xu et al. [5] was used to analyze the regions selected in Step 1 in the family data. This method allows testing multiple markers simultaneously without haplotype reconstruction, and provides significance levels. In brief, the FBAT-LC test proposed by Xu et al. [5] is based on a linear combination of single-marker FBAT test statistics using data-driven weights, where marker weight derivation is based on the "conditional mean model" [8]. The FBAT test for each bi-allelic marker is carried out for only one allele. When assuming an additive model, this test does not depend on the selected allele. Finally, for the *p*-values obtained for all candidate regions, different corrections for multiple testing were compared: no correction, Benjamini and Hochberg correction, and Bonferroni correction. A region was considered significant if the corrected *p*-value was less than 5%.

### Performance of the multiple-marker two-step strategy and comparison with the single-marker approach

We assessed the ability of our strategy to reveal regions containing the trait loci by comparing the results obtained from the analysis of all Problem 3 case-control and family data replicates with the answers that were provided. Because the local score was applied to case-control data in Step 1 and FBAT-LC to ASP data in Step 2, we formed 50 replicates of association-study data sets, each set being made of two independent samples: one replicate of case-control data and one replicate of family data. Each case-control data replicate included 1500 cases (one case drawn at random from each ASP) and 2000 controls genotyped for the 9187 SNPs. Each family data replicate included 1500 ASPs genotyped for all SNPs belonging to the candidate regions selected in Step 1.

To evaluate the performance of our strategy, we first identified, in each replicate, the true positive and the true negative regions among those selected in Step 1, a region being defined as positive if it contained at least one of the two flanking markers of any hidden trait locus. We then derived the three following quantities: 1) sensitivity, which is the proportion of true-positive regions that were correctly identified by FBAT-LC test; 2) specificity, which is the proportion of true-negative regions that were correctly identified by FBAT-LC test; 3) the false-discovery rate (FDR), which is the proportion of false positives among the declared significant results. An average estimate and standard deviation of each of these three quantities were computed over the 50 replicates of family data. The estimates of these quantities were compared according to the correction applied to the FBAT-LC *p*-values. The average proportion of trait loci detected by our two-step approach over the 50 replicates of association-study data sets was also derived.

We then conducted a two-stage single-marker analysis to be compare with our multiple-marker strategy. All 9187 genotyped SNPs were ranked according to the *p-*values associated with the Pearson chi-square test applied to the case-control genotypic contingency table. A number, *M*, of markers with the smallest *p*-values to be analyzed in Step 2 was selected. *M *was equal to the average number of markers belonging to the regions selected by the local score method over 50 replicates. In Step 2, a single-marker FBAT was applied to each of the *M *selected markers and *p*-values were either not corrected or corrected using either Benjamini and Hochberg or Bonferroni corrections. To be comparable with the above definition of a true-positive region, true-positive markers among the *M *selected markers were those flanking each trait locus. Estimates of the same performance indicators, as defined above, were derived over the 50 replicates of association-study data sets.

## Results

In Step 1, the local score method revealed an average of 381 regions (standard deviation (sd) = 7.79) with positive scores. These regions contained 472 SNPs on average (sd = 3.30). The distribution of the number of SNPs per region showed that Region 1 contained 38 markers on average, Regions 2 to 6 had more than 2 SNPs and up to 4 SNPs on average, the next 18 regions contained 2 SNPs, and the remaining ones had only 1 SNP.

As an illustration, Table 1 presents the outcomes of each step of our multiple-marker approach in the first association-study data set for 10 regions having the highest local scores in Step 1. Five of these regions were significant at the nominal 5% level in Step 2 and four of them remained significant after Bonferroni correction. These four regions were on chromosomes 6 (two regions), 11, and 18. The first region on chromosome 6 contained the DR, C and D loci, the second and third ones on chromosomes 11 and 18 contained the F and E loci respectively, while the other region on chromosome 6 did not harbor any locus involved in RA, and was therefore a false positive. We noted that this pattern of results was actually similar across all replicates, the first three regions (chromosomes 6, 11, and 18) always having the highest local scores in Step 1.

**Table 1:** Results for the first 10 regions in the first replicates

	Step 1				Step 2		
			
Region	Chr	IDs of the 2 extreme markers bounding a region		Local Scores^a^	FBAT_LC *p*-value^b^	Bonferroni corrected *p*-value	Trait loci in the region
1	6	128	162	8235.4874	0.000000	0.000000	DR, C, D
2	11	387	396	202.3407	0.000000	0.000000	F
3	18	269	269	44.5575	0.000000	0.000000	E
4	18	10	11	28.2714	0.042545	0.425450	
5	10	355	357	16.8203	0.476407	1.000000	
6	4	347	348	11.7416	0.440749	1.000000	
7	6	355	355	11.6840	0.002913	0.029130	
8	3	58	58	11.5742	0.137617	1.000000	
9	8	392	392	11.5131	0.387798	1.000000	
10	7	94	95	11.4852	0.706698	1.000000	

^a^Local Scores obtained from the first replicate of case-control data.

^b^*p*-Values obtained from FBAT-LC applied to regions selected in Step 1 in the first replicate of family data.

We first evaluated the performance of our strategy by selecting at the first step 50 regions with the highest local scores, which included 115 SNPs on average, thus representing 1% of the 9187 genotyped SNPs. Table 2 shows the average sensitivity, specificity, and FDR of the FBAT-LC statistic applied to these 50 regions and of the single-marker FBAT applied to each of the 115 SNPs having the lowest *p*-values among all genotyped SNPs. The average sensitivity was very high (>94%), regardless of *p*-values correction and analysis of regions or single markers. The average specificity was higher when examining regions (always greater than 95%) than single markers (78% without correcting *p*-values and 87% with correction). The FDR associated with FBAT-LC test decreased dramatically from 37% without correcting *p*-values to 3% when using a correction, while the FDR associated with single-marker FBAT remained higher than 74% even when *p*-values were corrected for multiple testing. We then examined all regions with positive local scores and found similar performance (results not shown). Moreover, the average proportion of trait loci detected by either the multiple-marker or single-marker two-step strategy was close to 55%: the DR, C, D, and E loci affecting RA risk directly and the F locus as a QTL for IgM were detected in at least 98% of the replicates, while the influence of Loci G and H on disease severity and the involvement of Loci A and B in more complex gene × gene (G × G) and gene × environment interactions (G × E) were almost never revealed. These results could be explained by the fact that the local score statistic was based on the Pearson chi-square test of association between genotype frequencies and affection status, and hence not dedicated to study severity or interactions.

**Table 2:** Comparison of multiple marker and single marker strategies

	Correction for multiple testing	Sensitivity^a^	Specificity^a^	FDR^a^	% of all TL detected^b^
		FBAT-LC			
			
Multiple markers^c^	None	0.97 (0.08)	0.95 (0.03)	0.37 (0.17)	0.56 (0.02)
	Benjamini & Hochberg	0.95 (0.10)	0.99 (0.09)	0.03 (0.09)	0.55 (0.02)
	Bonferroni	0.94 (0.11)	0.99 (0.09)	0.03 (0.09)	0.55 (0.03)
					
		Single-marker FBAT			
			
Single marker^d^	None	0.97 (0.09)	0.78 (0.03)	0.83 (0.02)	0.56 (0.02)
	Benjamini & Hochberg	0.95 (0.11)	0.87 (0.01)	0.74 (0.02)	0.55 (0.02)
	Bonferroni	0.95 (0.11)	0.87 (0.01)	0.74 (0.03)	0.55 (0.02)

^a^Mean (standard deviation) of sensitivity, specificity, and false-discovery rate of FBAT-LC (multiple markers) and single-marker FBAT computed over 50 replicates of family data.

^b^Mean (standard deviation) of the proportion of all trait loci (TL) selected in Step 1 and detected in Step 2 by FBAT at the 5% level over 50 replicates.

^c^50 regions were selected in Step 1 using the local score method.

^d^115 SNPs were selected in Step 1 (see text) using the Pearson chi-square applied to case-control genotypic contingency table.

We repeated our two-step analysis by using other test statistics to compute the local score in Step 1. Regarding severity, we used the test of Spearman rank correlation between marker genotype (0, 1, 2) and severity (from 1 to 5). An average of 395 regions (containing 467 markers on average) with positive local scores was found. Applying the same selection criterion as before, we kept 50 regions with the highest local scores to be analyzed in Step 2. The performance of FBAT-LC is shown in Table 3. Both sensitivity (96% or more) and specificity (99%) were very high and the FDR was 14% when *p-*values were corrected. Moreover, the two loci influencing RA severity were detected in at least 87% of replicates. Regarding the loci involved in G × G and G × E interactions, we used a logistic regression model incorporating either one of these interactions and the likelihood-ratio test of interaction served as input for computing the local scores. However, there was no improvement in the detection of Loci A and B.

**Table 3:** Performance of the multiple-marker strategy in disease severity analysis

Correction for multiple testing	FBAT LC			% of severity loci detected^b^
		
	Sensitivity^a^	Specificity^a^	FDR^a^	
Without correction	1.00 (0.00)	0.92 (0.03)	0.75 (0.13)	0.91 (0.19)
Benjamini & Hochberg	0.96 (0.20)	0.99 (0.01)	0.14 (0.24)	0.87 (0.26)
Bonferroni	0.96 (0.20)	0.99 (0.01)	0.14 (0.24)	0.87 (0.26)

^a^Mean (standard deviation) of sensitivity, specificity and false-discovery rate of FBAT-LC test computed over 50 replicates of family data when 50 regions were selected in Step 1.

^b^Mean (standard deviation) of the proportion of severity loci selected in Step 1 and detected in Step 2 by FBAT at the 5% level over 50 replicates when 50 regions were selected in Step 1.

## Discussion

Overall, our results show that the present two-step strategy based on sets of markers provides significant evidence for all four loci affecting RA risk (DR, C, D, E), one QTL for IgM (F) and two loci influencing RA severity in almost all replicates, provided appropriate test statistics are used in Step 1 to compute the local scores. These regions were always detected at the first step for the five former loci and in 87% of replicates for the two severity loci. All of these regions were confirmed at least 94% of the time in Step 2. This shows the efficiency and flexibility of this overall strategy, which can use different test statistics within the same framework. However, loci involved in more complex interactions (A, B) were difficult to identify, which may be partly due to the relatively small importance of these interactions and/or weak linkage disequilibrium of these loci with the analyzed markers.

When comparing the proposed multiple-marker strategy to the single-marker approach, these two strategies showed similar power to detect RA loci and had both high sensitivity and specificity. However, while the FDR associated with the multiple-marker FBAT-LC decreased significantly when *p*-values were corrected for multiple testing, the FDR associated with the single-marker FBAT remained high. Previous simulations had shown that the local score statistic was more powerful than the single-marker approach in case-control data [7]. The present findings may be partly due to the generated model in which several loci, especially those on chromosome 6, played an important role in the disease and were thus likely to be always detected. Thus, further comparisons of multiple and single-marker-based methods in other data sets generated under different models appear warranted.

The results presented here were obtained for the first 50 selected regions with highest local scores in Step 1, which were followed up in Step 2. However, varying the number of selected regions from 10 regions to all regions with positive local scores had a small impact on sensitivity, specificity, and FDR as well as on proportion of TLs detected. This shows that the performance of the proposed strategy was already satisfactory even for a small number of selected regions. However, this may be at least partly due to the strong effect of most TLs on RA. Nevertheless, selecting a small number of regions (50, or as few as 10 regions) in Step 1 might be an appropriate strategy that can minimize the multiple testing problem, although disease models other than the one simulated here need to be explored before drawing a definite conclusion.

We used a two-stage analytical approach using two different statistical methods applied to two independent data sets. However, in the context of a two-stage design for genome-wide association studies, Skol et al. [1] have shown that the joint analysis of the two steps was more efficient than the independent analysis of each step, this analysis being based on single-marker tests of marker allele frequencies in case-control data. Comparison of this latter strategy to the one proposed here would be worth conducting, but the framework of this comparison needs to be further defined.

We used here the local score method as a simple screening tool in a two-stage design. However, this approach can also stand on its own in genome-wide association studies. The significance of local scores can be determined via the extreme values theory. Indeed, under the null hypothesis (*H*_0_), the local score is known to follow the GUMBLE distribution asymptotically. However, this asymptotic approximation is only valid under linkage equilibrium, which generally does not hold. A Monte-Carlo simulation-based version taking these dependencies into account has been implemented (available at ), but simulations increase the time of execution notably. The simple use of the local score method to rank regions to be further tested in another data set, as proposed here, was fast to run because the overall two-step strategy took less than 10 minutes to analyze one sample of 2000 cases/1500 controls in Step 1 and one sample of 1500 affected sib pairs in Step 2. Finally, the proposed strategy is also flexible because it allows different types of data and different test statistics at each step to be considered. Use of a case-control sample in Step 1 might be preferred because it requires less cost and less time to collect data [9], and using a family-based method in Step 2 protects against population stratification.

## Conclusion

The proposed two-step multiple-marker strategy provides a general and flexible framework for genome-wide association studies that can integrate different types of data and different test statistics. By considering regions instead of single markers, this strategy minimizes the multiple testing problem and the number of false-positive results. It is also simple and fast to run. Evaluation of this strategy in more complex situations than the ones examined here and extensive comparison with other strategies proposed for genome-wide association studies would be worth performing.

## Competing interests

The author(s) declare that they have no competing interests.

**Figure 1:**
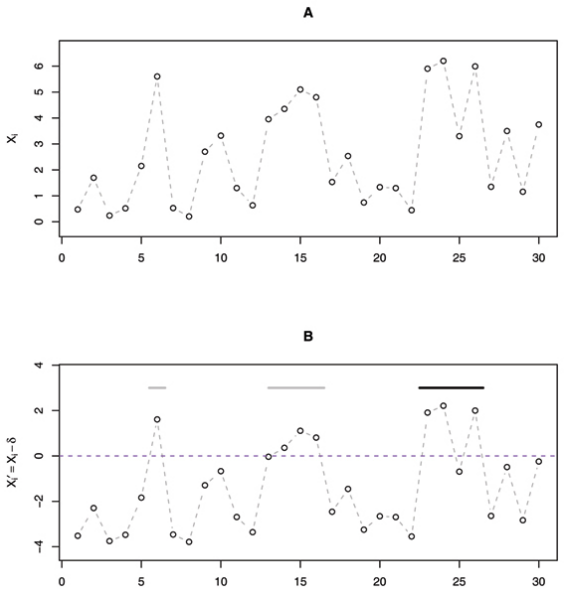
**Schematic representation of the local scores method**. A, scores *X*_*i *_(e.g., Pearson chi-squared statistics for case-control genotype frequencies) at marker locations along a chromosome. B, Scores ${X^{\prime }}_{i}$ at marker locations along a chromosome; ${X^{\prime }}_{i}$ = *X*_*i *_- *δ*, *δ *being the value of statistic *X*_*i *_at the 5% level.
